# Clinical Relevance of Pharmacogenetics in Serotonin Syndrome

**DOI:** 10.1155/2020/8860434

**Published:** 2020-10-07

**Authors:** Dehuti Pandya, My Tran, Monica Verduzco-Gutierrez

**Affiliations:** ^1^Department of Pharmacy, TIRR Memorial Hermann Hospital, 1333 Moursund St., Houston TX 77030, USA; ^2^University of Texas at Austin, College of Pharmacy, 2409 University Ave., Austin TX 78712, USA; ^3^Department of Rehabilitation Medicine, Joe R. and Teresa Lozano Long School of Medicine at UT Health San Antonio, 7703 Floyd Crule Dr Mail code: 7798, San Antonio, TX 78229, USA

## Abstract

Serotonin syndrome is a predictable life-threatening condition that is caused by serotonergic stimulation of the central and peripheral nervous systems. A patient's genetic profile can amplify exposure risk as many serotonergic drugs are metabolized by CYP450 enzymes, and these enzymes may be altered in functionality. We report a case of an elderly man who presented with serotonin syndrome after a dose change in valproic acid 5 weeks prior. His medication list consisted of low-dose serotonergic agents, which is unusual as most cases of serotonin syndrome involve higher doses. A review of his pharmacogenetic profile is presented to retrospectively evaluate the additive risk for serotonin syndrome and implications on resuming serotonergic agents.

## 1. Introduction

Serotonin syndrome (SS) is a potentially life-threatening, drug-related condition precipitated by increased serotonergic activity in the central and peripheral nervous system. Patients with SS present with any symptom from the clinical triad of mental status changes, autonomic hyperactivity, and neuromuscular abnormalities [1]. SS can occur in all age groups, and higher risk is associated with increased use of serotonergic agents such as antidepressants, monoamine oxidase inhibitors, and opioids [2]. Management approaches include discontinuation of these agents, benzodiazepines for sedation, supportive care, and cyproheptadine for serotonin antagonism [1].

Pharmacogenetics has been used in recent years to study how genes affect a person's medication response. The gene variants contribute to variability in the drug concentration which is responsible for efficacy and toxicity. A study by Tansey et al. found that common genetic variants explain 42% of individual differences in antidepressant response [3]. Antidepressants are the main risk factor for serotonin syndrome [2]. Applying genetic testing can improve medication outcomes, prevent such serious toxicity, and achieve medical cost savings. Pharmacogenetic-based guidelines/algorithms and a number of valid pharmacogenetics decision support kits have been developed to optimize treatment selection [4].

However, to date, clinical evidence regarding the use of pharmacogenetic results to manage patients who have experienced SS is still limited. We report a case of SS from low-dose serotonergic agents and the application of pharmacogenetics in individualizing treatment options for the patient.

## 2. Case Description

**Figure 1:**
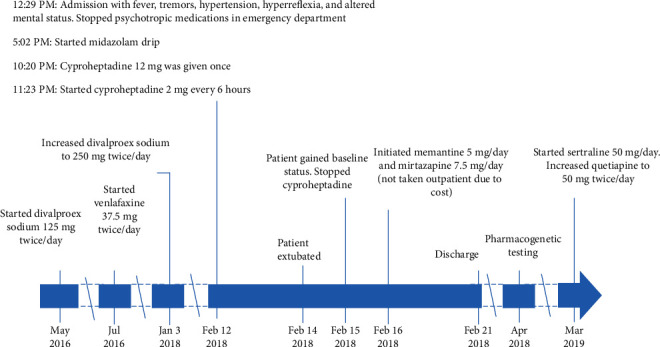
Timeline of patient's clinical course.

A 74-year-old 75 kg male presented to the emergency department with generalized shaking, tremors, clonus, and altered level of consciousness. The patient was last seen normal nine hours prior to arrival by his spouse. His past medical history included diabetes mellitus type II and traumatic brain injury (TBI), with a subdural frontal and temporal intracranial hemorrhage, incurred after falling from a ladder 3 years prior. He had no history of seizure disorder. Home medications on admission were divalproex sodium 250 mg twice/day, quetiapine 25 mg twice/day, venlafaxine 37.5 mg twice/day, trazodone 50 mg as needed (taken 2-3 times/week), donepezil 10 mg once/day, insulin glargine 15 unit once/day, melatonin 3 mg once/evening, magnesium oxide 400 mg once/day, metformin 1000 mg twice/day, pravastatin 20 mg once/evening, and tamsulosin 0.4 mg once/evening. The patient's clinical course is summarized in Figure 1.

On admission, the patient's vital signs were maximum temperature of 39.7°C, heart rate of 120 beats per minute, blood pressure of 188/84 mmHg, and respiration rate of 23. Abnormal labs included elevated creatinine kinase level of 6.34 and hypocalcemia of 6.8. Clinical examination revealed tremors, 3+ hyperreflexia in lower limbs, and Glasgow Coma Scale of 3. He was intubated and immediately transferred to the medical intensive care unit. His hyperthermia was unresponsive to antipyretics and cooling blankets. Several etiologies for encephalopathy—hepatic, endocrine, metabolic, Wernicke's, seizures—were ruled out through laboratory, diagnostic, and microbiological testing. Viral and bacterial encephalopathy remained in the differential diagnosis, but was of low suspicion due to an unremarkable cerebrospinal fluid analysis. Although the patient was at risk for seizure disorder due to his history of TBI, the neurology team conveyed a low level of suspicion for seizures due to a negative electroencephalogram (EEG) and a lack of tonic or clonic activity.

Serotonin syndrome was of high suspicion as evidenced by the patient's home medication list that included divalproex sodium, venlafaxine, and trazodone. The patient suffered from depression, agitation, aggression, migraines, and sudden mood swings resulting from the aforementioned TBI for which he took venlafaxine 37.5 mg twice/day, trazodone 50 mg 2-3 times/week, quetiapine 25 mg twice/day, and divalproex sodium 250 mg twice/day. Quetiapine and trazodone were initiated 3 years ago immediately following the patient's TBI. The patient was originally prescribed escitalopram, but was switched to venlafaxine due to persistent depressive symptoms and migraine prophylaxis twenty months prior to this admission. Divalproex sodium was started at 125 mg twice daily for migraine prophylaxis and mood swings eighteen months prior to this admission. Divalproex sodium was then increased to 250 mg twice/day 2 months prior to this admission.

The neurology team started the patient on cyproheptadine 12 mg once and then 2 mg every 2 hours and midazolam drip for serotonin syndrome within 24 hours of admission. All psychotropic medications including quetiapine, divalproex sodium, donepezil, trazodone, memantine, venlafaxine, and melatonin were held. As the patient regained baseline neurological and muscular functioning on day 3, he was extubated, and cyproheptadine was discontinued after a total of 50 hours of therapy. After a psychiatric consultation, trazodone and venlafaxine were permanently discontinued. The patient was discharged on mirtazapine 7.5 mg for insomnia and mood, memantine 5 mg for cognition, and quetiapine 25 mg twice daily for management of his aggression. Divalproex sodium was to be resumed outpatient at the discretion of his primary care physician.

The patient followed up with his physical medicine and rehabilitation (PM&R) physician 2 months after his hospital admission. He was unable to continue mirtazapine after discharge due to prohibitive cost. His spouse endorsed worsening depressive and neurobehavioral symptoms following his medication changes. The patient reported more frequent migraines and diminished quality of sleep. The patient's physician ordered a pharmacogenetic test through the clinic pharmacy services. The results indicated that the patient was an intermediate metabolizer of venlafaxine due to the presence of a *CYP2D6*∗*4/*∗*41* polymorphism. Different enzymes that metabolized each of the drugs that the patient was taking were also examined. Trazodone and quetiapine are metabolized by CYP3A4. Divalproex sodium is primarily metabolized by glucuronosyltransferase UGT (30-50% of dose) and different CYP isoforms (10% of dose) including CYP2A6, CYP2B6, CYP2C9, and CYP3A4 [5]. Other genes relevant to mental health disorders such as *CYP2C9*, *CYP2C19*, *CYP2B6*, *CYP3A4*, *CYP3A5*, *UGT2B15*, *COMT*, *HTR2C*, and *DRD2* are included in Table 1. Using this pharmacogenetic report, sertraline 50 mg once/day was initiated, and quetiapine was increased from 25 mg to 50 mg twice/day to better manage depression and aggression without SS recurrence.

## 3. Discussion

According to the Hunter Criteria Decision Rules for SS, patients must meet one of the requirements listed in Table 2 with recent use of serotonergic agents within the past five weeks [6].

This case fulfilled multiple symptoms consistent with SS including tremor, hyperreflexia, fever of 39.7°C, and confusion. Neuromuscular malignant syndrome (NMS) is also on the differential diagnosis due to overlapping vital sign changes and altered mental status. However, NMS has hypoactive reflexes and results from the use of dopaminergic agents [1]. This patient received 3 agents that have been closely associated with SS. Data has reported multiple SS cases with venlafaxine, trazodone, and valproate, especially in drug combinations [7–9]. Our patient had been stable on these medications for at least 18 months; however, the dose increase in divalproex sodium from 125 mg to 250 mg one month prior to admission may have triggered the accumulated serotonergic effect of his drug regimen. SS usually has quick onset after initiation, change, or overdose of medications [1]. Although almost 60% of patients have presenting symptoms within six hours of drug exposure, there are previous cases reporting delayed onset of SS. Ong et al. mentioned an onset of up to one year in a 69-year-old patient on vortioxetine 10 mg standard dose [10]. A recent case in 2019 also noted a delayed 4-month onset with venlafaxine uptapering to 75 mg in combination with tianeptine, methylphenidate, ropinirole, carbidopa/levodopa, and bromocriptine [11]. In this case, we reported the delay 1-month onset with increasing divalproex sodium to 250 mg twice daily with concomitant use of venlafaxine and trazodone. Since the patient was not on high doses of these serotonergic agents, it is reasonable to postulate, given his pharmacogenetic profile, that the patient slowly accumulated the drugs.

Among the three serotonergic agents on the patient's regimen, venlafaxine has the most data associated with SS and pharmacogenetic research [1]. It is a serotonin-norepinephrine reuptake inhibitor indicated for depression, migraine prophylaxis, generalized anxiety, and among other psychiatric disorders. CYP2D6 is the major hepatic enzyme involved in the first-pass metabolism of venlafaxine to synthesize the active metabolite, O-desmethylvenlafaxine. Up to 82% of the drug is excreted renally as metabolites [5]. Based on the test results, our patient is characterized as a *CYP2D6*∗*4/*∗*41* heterozygote. Over 120 variations have been identified for CYP2D6 with phenotype consequences ranging from increased function to normal function or complete loss of function [12]. Allele ∗4 has the nucleotide guanine substituted by adenine at position 1847 (1847G > A). This single-nucleotide polymorphism alters an mRNA splicing site that leads to a frameshift and early translation termination. Thus, allele ∗4 results in the nonfunctional CYP2D6 protein. There are other major mutant alleles that are poor metabolizers (PM) like ∗4, and they are alleles ∗3,∗5, and ∗6. Next, allele ∗41 also causes alternative splicing due to adenine at position 2989 instead of guanine (2989 G > A). This change does not cause frameshift; thus, the CYP2D6 protein product still retains function but at decreased level [12]. Taken together, our patient's CYP2D6 phenotype is an intermediate metabolizer (IM). Race is a factor in the occurrence of gene variability, and CYP2D6 enzyme is a good example of this. The prevalence of individuals having this phenotype is about 2-11%, generally presenting more in the Caucasian population than other ethnic groups such as Asians and Africans [13]. Furthermore, venlafaxine is further metabolized by minor pathways via CYP2C19, CYP2D6, and CYP3A4 to less active metabolites to be excreted [7]. This patient is also a CYP3A4 intermediate metabolizer with the genotype ∗*1/*∗*22* (Table 1). Allele ∗22 indicates a change from cytosine to thymine at position 15389 (15389C > T) that alters splicing and decreases the mRNA expression and enzyme activity [14]. Similar process occurs with his *CYP2C19*∗*1/*∗*2* polymorphism. Allele ∗2 has 12662A > G, 19154G > A, and 80161A > G to alter splicing and make the allele nonfunctional [12]. Due to decreased activity of IM's CYP2D6, CYP2C19, and CYP3A4, higher plasma concentration of venlafaxine may increase the probability SS [7]. Overall, his pharmacogenetic profile predisposes the patient to more venlafaxine toxicity even at low dosage.

We suspect that divalproex sodium was another factor in the development of SS as symptoms onset occurred after increment of valproate dose. Valproate is an antiepileptic compound that is used for many disorders including epilepsy, seizures, migraine prophylaxis, and bipolar. Valproate has been linked to SS when it is initiated in patients who were on amitriptyline and psychiatric agents such as lithium, risperidone, and olanzapine [8, 15]. Valproate's antiepileptic effect could be due to enhancing GABA-mediated inhibition in the CNS, while its serotonergic effect results from decreased serotonin reuptake [16]. Glucuronidation by uridine 5′-diphospho-glucuronosyltransferase (UGT) and beta-oxidation in the mitochondria metabolize up to 50% and 40% of dose, respectively, and CYP450 accounts for the remaining 10% [5]. Most pharmacogenetic data discussed the UGT1A6 polymorphism impact on dosage. Chung et al. found that carriers of UGT1A6 with 19 T > G, 541A > G, and 552A > C required higher valproate dose in a study on 162 seizure patients [17]. Another study by Krishnaswamy et al. showed that the ∗2 allele was associated with more glucuronidation of valproate than the ∗1 allele [18]. Knowing the genotype and phenotype of UGT enzymes are beneficial in predicting patient outcomes in response to valproate. We only have UGT2B15 normal metabolizer phenotype, thus, does not explain the SS effect observed in our patient. More testing on other UGT enzymes is warranted to establish the pharmacogenetic predisposition. In terms of CYP450 allele, *CYP2A6*∗*4*, *CYP2B6*∗*6*, and *CYP2C9*∗*3* are associated with higher mean valproate concentrations compared to wild-type in a study of 179 Asian patients with epilepsy [19]. Substantially, decreased valproate oxidation metabolism was also reported for *CYP2C9*∗*2* allele in human liver microsomes, although in vivo data is limited [20, 21]. Our patient is an intermediate metabolizer for both *CYP2B6* with ∗*1*/∗*6* genotype and *CYP2C9* with ∗*1*/∗*2* genotype. *CYP2B6*∗*6* is defined by two single-nucleotide polymorphisms 15631G > T and 18053A > G that cause aberrant splicing and low protein expression. *CYP2C9*∗*2* has 3608C > T that alters the active site and reduces catalytic efficiency [12]. Collectively, these alterations may contribute to increased risk for valproate toxicity in our patient.

Trazodone is another agent on patient's medication list with serotonergic activity based on the mechanism of action. It represents atypical antidepressant that selectively inhibits reuptake of serotonin and acts as serotonin receptor antagonist [22]. SS has been reported in patients taking trazodone 50-300 mg/day in combination with SSRIs and MAOIs [9, 23]. Trazodone is mainly metabolized to active metabolite mCPP by CYP3A4. CYP2D6 further hydroxylates mCPP to produce OH-mCPP that is excreted via phase 2 metabolism [22]. Pharmacogenetic research for trazodone is limited in comparison to previous two agents. Unlike venlafaxine and valproate, there is currently no genes clinically annotated for trazodone in PharmGKB and CPIC databases. No significant study has linked CYP3A4 polymorphisms with trazodone pharmacokinetics. For CYP2D6, a study on 54 depressed Japanese patients taking trazodone concluded that CYP2D6 genotypes cannot predict the steady state concentrations of either trazodone or mCPP [24]. Contrary to the expectation that normal metabolizer has less toxicity than poor metabolizer, a more recent study by Saiz-Rodriguez et al. showed increased likelihood of dizziness when treated with trazodone in CYP2D6 normal metabolizer compared to poor metabolizer and intermediate metabolizer [25]. These data suggest little role for CYP2D6 pharmacogenetic in explaining SS associated with trazodone.

Quetiapine and donepezil were also discontinued at admission due to psychotropic activity. Quetiapine demonstrates efficacy for treatment of schizophrenia, bipolar, and depression via antagonism of dopamine, serotonin, and norepinephrine receptors. CYP3A4 is the primary enzyme responsible for quetiapine metabolism in vitro and vivo while CYP2D6 plays a small role in 7-hydroxylation of quetiapine to produce active metabolite in vivo [26]. IM phenotypes of these two enzymes may accumulate plasma concentration. A case report in 2006 mentioned increased adverse drug reactions in clomipramine and quetiapine combination for a patient with CYP2D6 poor metabolizer and low CYP3A4/5 activity status [27]. Quetiapine along with other second-generation antipsychotics such as lurasidone and ziprasidone has been associated with SS using the FDA Adverse Event Reporting System [28]. Thus, our patient is also predisposed to quetiapine toxicity considering his genetic finding. Last but not least, donepezil is an acetylcholinesterase inhibitor indicated for Alzheimer's disease. Donepezil is metabolized by both CYP3A4 and CYP2D6, so similar effects are expected as in quetiapine. In fact, FDA-approved drug label for donepezil emphasized that there are differences in clearances between CYP2D6 poor, extensive, and ultra-rapid metabolizers affecting drug efficacy. Donepezil is noted to cause neuroleptic malignant syndrome which is a common differential diagnosis, yet there has been SS case reports as well [29]. Liu et al. described a SS case in a patient taking normal doses of SSRI with olanzapine and donepezil [30]. Similar to quetiapine, although donepezil did not directly cause symptoms in this patient, his genetic makeup could certainly play an important role in increasing the patient's susceptibility to SS with his drug regimen.

After discharge, the patient's revised antipsychotic regimen included sertraline and quetiapine. He has not had a recurrence of SS with his new regimen. Sertraline was selected based on the pharmacogenetic profile. Hepatic metabolism via multiple CYP450 plays a main role in sertraline metabolism based on vitro studies [31]. In vivo and pharmacogenetic studies emphasize on CYP2C19 impact on sertraline pharmacokinetics. Patients carrying *CYP2C19*∗*2* have slower sertraline metabolism rate than CYP2C19 normal metabolizer [32]. As a CYP2C19 intermediate metabolizer, our patient can accumulate more toxicity exposure. However, the CPIC guideline has a strong recommendation to safely maintain normal starting dose of SSRIs in this patient population with higher level of evidence than in the case of venlafaxine and CYP2D6 [13]. Thus, switching from venlafaxine to sertraline can reduce SS risk. All other SSRIs can be used like citalopram, fluoxetine, and vortioxetine with the consideration that there may be dose limits or starting at lower doses due to CYP2D6/2C19 limited functioning. Duloxetine is extensively metabolized by CYP2D6 and CYP1A2, and European Medicines Agency says limited data suggest that concentrations of duloxetine are higher in CYP2D6 poor metabolizers. Our patient is an intermediate metabolizer, so presumably he can use the agent with close monitoring just like the SSRIs. Other agents like tricyclics (amitriptyline, doxepin, nortriptyline) metabolized by CYP2D6 or CYP2C19 would require lower dosing and close monitoring for side effects like SS and pose a higher risk than SSRI. Decreasing the number of antipsychotic drugs from 5 agents to 2 agents also significantly minimizes toxicity in our patient.

**Figure 2:**
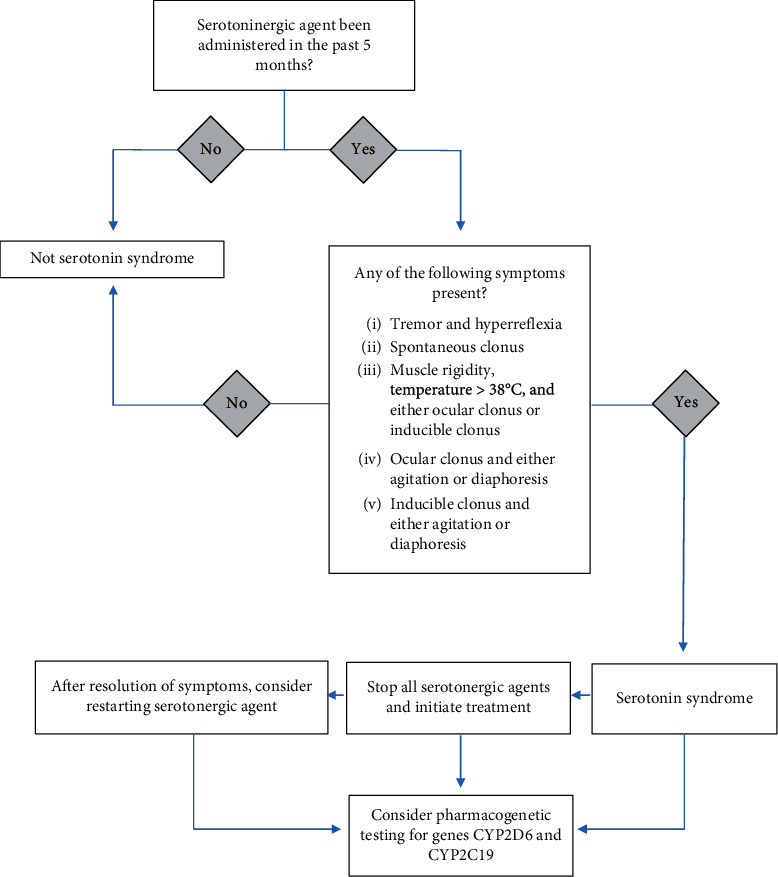
Algorithm for incorporating pharmacogenetic testing in the management of serotonin syndrome. Diagnosis criteria adapted from Dunkley et al.

Pharmacogenetic testing is being utilized to guide therapy optimization and to improve patient safety and cost savings. It offers great benefits by facilitating the healthcare team in selection, initiation, and adjustment of a drug regimen. Patient's therapy is individualized via incorporating the clinically actionable suggestions provided by the report. For our patient, multiple agents are linked to CYP2D6 metabolism, thus limiting the number of agents that could have been used that do not clinically get affected by the altered metabolism. Besides CYP2D6, the report also provided us information on other genes related to psychiatric conditions as noted in Table 1; however, lack of evidence-based clinical suggestions for these genes remains the biggest limitation of pharmacogenetic testing. Currently, expert consensus reviews agreed that only 4 genes, *CYP2D6*, *CYP2C19*, *HLA-A*, and *HLA-B,* have sufficient supporting evidence to be deemed clinically actionable in psychopharmacology. An evidence level of A or B in CPIC suggests actionable prescribing based on genetics should be considered. Applied to our patient, CPIC assigned an evidence level of B for the drug-gene interaction between *CYP2D6*-venlafaxine and level B/C for *CYP2D6*-donepezil. Discontinuing both agents was a reasonable decision. *CYP2D6* and *CYP2C19* are the mainstay of antidepressants while the *HLA* gene is related to anticonvulsants such as carbamazepine and oxcarbazepine [33]. In general, any psychiatry panel should include these genes as a minimum requirement. Thus, for patients with SS, we propose an algorithm to follow up after the diagnosis to include *CYP2C19* and *CYP2D6* assessment as included in Figure 2. Although other genes are provided, clinicians should evaluate literature, utilize reliable resources such as the CPIC guideline and PharmGKB database, and assess the patient's complete picture to make an informed use of the pharmacogenetic findings.

In summary, SS risk is greater with concomitant use of serotonergic agents. Genetic predisposition further increases the risk at standard dosages by affecting drug pharmacokinetics via single nucleotide polymorphisms in metabolic enzymes. The SS onset can be delayed in these individuals as seen in our patient due to slow drug accumulation. Recognizing this clinical picture and using an algorithm similar to the one we used in our case have great implications on future serotonergic agents initiated on these patients. Utilizing the *CYP2D6* and *CYP2C19* gene testing can help identify higher risk populations and associated agents in psychiatric pharmacotherapy.

## Figures and Tables

**Table 1:** Patient's pharmacogenetic tests done with genotype and phenotype results.

Gene	Genotype	Phenotype
*CYP3A4^a^*	∗*1/*∗*22*	Intermediate metabolizer
*CYP3A5^b^*	∗*1/*∗*3*	Intermediate metabolizer
*CYP2B6^c^*	∗*1/*∗*6*	Intermediate metabolizer
*CYP2C9^d^*	∗*1/*∗*2*	Intermediate metabolizer
*CYP2C19^e^*	∗*1/*∗*2*	Intermediate metabolizer
*CYP2D6 ^f^*	∗*4/*∗*41*	Intermediate metabolizer
*COMT^g^*	*Val158Met A/A*	Low COMT activity
*HTR2C^h^*	*-759C > T C/C*	Homozygous for the C allele (rs3813929)
*HTR2C^h^*	*114138144C > G C/C*	Homozygous for the C allele (rs1414334)
*DRD2^i^*	*Taq1A A/G*	Altered DRD2 function
*UGT2B15^j^*	∗*1/*∗*1*	Normal metabolizer

^a^Cytochrome P450 family 3 subfamily A member 4. ^b^Cytochrome P450 family 3 subfamily A member 5. ^c^Cytochrome P450 family 2 subfamily B member 6. ^d^Cytochrome P450 family 2 subfamily C member 9. ^e^Cytochrome P450 family 2 subfamily C member 19. ^f^Cytochrome P450 family 2 subfamily D member 6. ^g^Catechol-O-methyltrasferase. ^h^5-Hydroxytryptamine receptor 2C. ^i^Dopamine receptor D2. ^j^Glucuronosyltransferase family 2 member B15.

**Table 2:** Hunter criteria for serotonin syndrome.

Use of serotonergic agent and one of the following
(1) Spontaneous clonus (2) Inducible clonus and agitation OR diaphoresis (3) Ocular clonus and agitation OR diaphoresis (4) Tremor and hyperflexia (5) Hypertonic and temperature 38°C and ocular clonus or inducible clonus

## Data Availability

Not declared.
